# A Time-Dependent Hierarchical Model for Elastic and Inelastic Scattering Data Analysis of Aerogels and Similar Soft Materials

**DOI:** 10.3390/gels8040236

**Published:** 2022-04-12

**Authors:** Cedric J. Gommes

**Affiliations:** Department of Chemical Engineering, University of Liège, B6C, Allée du Six Août 3, B-4000 Liège, Belgium; cedric.gommes@uliege.be

**Keywords:** silica aerogels, thermal fluctuations, small-angle scattering, neutron spin-echo, stochastic models, Boolean models, van-Hove correlation functions, porous materials

## Abstract

Soft nanomaterials like aerogels are subject to thermal fluctuations, so that their structure randomly fluctuates with time. Neutron elastic and inelastic scattering experiments provide unique structural and dynamic information on such systems with nanometer and nanosecond resolution. The data, however, come in the form of space- and time-correlation functions, and models are required to convert them into time-dependent structures. We present here a general time-dependent stochastic model of hierarchical structures, with scale-invariant fractals as a particular case, which enables one to jointly analyze elastic and inelastic scattering data. In order to describe thermal fluctuations, the model builds on time-dependent generalisations of the Boolean model of penetrable spheres, whereby each sphere is allowed to move either ballistically or diffusively. Analytical expressions are obtained for the correlation functions, which can be used for data fitting. The model is then used to jointly analyze previously published small-angle neutron scattering (SANS) and neutron spin-echo (NSE) data measured on silica aerogels. In addition to structural differences, the approach provides insight into the different scale-dependent mobility of the aggregates that make up the aerogels, in relation with their different connectivities.

## 1. Introduction

A distinctive characteristic of soft nanoscale materials is that they are subject to thermal fluctuations, and their structure therefore randomly fluctuates with time. Scattering experiments can provide invaluable information on the structure and dynamics of such systems, which can hardly be obtained by any other means [1]. Typically, elastic scattering of either X-rays (SAXS) or neutrons (SANS) provides structural information corresponding to an instantaneous snapshot of the fluctuating structure [2,3]. Inelastic scattering of thermal neutrons—as measured e.g., on neutron spin-echo (NSE) instruments [4]—provides dynamic information [3,5]. Unfortunately, the information from scattering data comes in the form of correlation functions, which is indirect and generally incomplete [6]. Significant data analysis is therefore required to convert scattering patterns into structural and dynamical insight. Moreover, in order to cope with the uncertainties inherent to data incompleteness, one generally has to rely on models.

In the present contribution, we propose a general time-dependent model useful for analyzing elastic and inelastic scattering data in materials with hierarchical aggregate structure. The developments are oriented towards the analysis of the fluctuating structure of aerogels at nanometer scale, but they are more general. A central characteristic of the model is that it aims at describing disordered structures, and this is achieved through a stochastic approach [7,8,9,10]. The use of stochastic models for analyzing disordered structures through elastic scattering goes a long way [11,12,13,14,15,16,17,18] but developments towards time-dependent structures and inelastic scattering have only started more recently [19]. The second general characteristic of the model is that it is hierarchical, which is a property shared by many materials [20,21]. In the particular case of aerogels, the hierarchy often takes the form of a scale-invariance fractal structure [22]. The model we propose offers a general framework to describe the scale-dependent structure and dynamics of hierarchical disordered systems, and to estimate the relevant structural and dynamical parameters from elastic and inelastic scattering data.

The structure of the paper of the paper is the following. The general formalism of stochastic structural models is introduced first, together with a discussion of the space- and time-dependent correlation functions relevant to elastic and inelastic scattering. The hierarchical model is presented afterwards. The static case is discussed first and particularized to scale-invariant fractal structures. The model is then made time-dependent by allowing the structural units to randomly move, either ballistically or diffusively. Finally, the model is applied in the discussion to analyze SANS and NSE data measured on silica aerogels by Schaefer et al. [23]. The results are analyzed in terms of the scale-dependent mobility of the aggregates that make up the aerogels.

## 2. Results and Discussion

### 2.1. General Formalism of Stochastic Structural Models, and Its Relation to Scattering

The structural models considered in this work are stochastic, which means they are defined through the probabilistic rules used to generate them [7,8,9,10]. In this context, it is customary to define the indicator function I(x) of the structure, which is a random function taking the value 1 when a given point x is in the solid phase and 0 if it is in the pore space. As we shall be concerned with time-dependent structures, we generalize this definition slightly as

(1) $$\begin{array}{c} I\left(x,t\right)=\left\{\begin{array}{cc} 1 & \mathrm{if}\mathrm{point}\;x\;\mathrm{is}\mathrm{in}\mathrm{the}\mathrm{solid}\mathrm{at}\mathrm{time}\;t\\ 0 & \mathrm{otherwise} \end{array}\right. \end{array}$$

As the value taken by I(x,t) for any given x and *t* is a random variable, it can only be characterised statistically.

In terms of the indicator function, the solid fraction of the material is defined as follows:

(2) $$\phi_{1}=\langle I\left(x,t\right)\rangle$$

where the brackets 〈.〉 stand for the average value. In principle, the average can be understood in three different ways. First, it can be seen as an ensemble average, calculated over all possible realisations of the model for a fixed x and *t*. It can also be seen as a space average, calculated over all possible values of x for a given realisation and a given time *t*. Finally, it can also be seen as a time-average, for a given realisation and a given position x. As the models we consider here are stationary in both space and time and ergodic [24], the three definitions are mathematically equivalent, and $\phi_{1}$ coincides with the classical and intuitive definition of a density.

The scattering properties of a system are expressed in terms of its covariance $C_{11}\left(r,\tau \right)$—occasionally referred to as a correlation function—defined as the probability for two points at distance r from one another to belong to the solid phase, with a time interval τ in between. In terms of the indicator function, this can be written as the following average:

(3) $$C_{11}\left(r,\tau \right)=\langle I\left(x,t\right)I\left(x+r,t+\tau \right)\rangle$$

where we have assumed that the structure is isotropic so that the dependence is only through the modulus r=|r|. The covariance is equal to $\phi_{1}$ for r=0 and τ=0. For the types of space- and time-stationary models that we are concerned with, $C_{11}\left(r,\tau \right)$ converges to $\phi_{1}^{2}$ for infinitely large values of either *r* or τ.

Both elastic and inelastic scattering properties of a system are controlled by the so-called intermediate scattering function, defined as the Fourier transform of the covariance, namely [3,5,25]

(4) $$I\left(q,\tau \right)=\int_{0}^{\infty }\frac{\sin[qr]}{qr}\left(C_{11}\left(r,\tau \right)-\phi_{1}^{2}\right)4\pi r^{2}dr$$

In particular, the value for τ=0 is relevant to the elastic scattering of say, X-rays or neutrons, such as SAXS or SANS [2,26]. This means that the latter techniques are blind to the time-dependence of the structure; in the case of systems at thermal equilibrium, they only provide an instantaneous snapshot of a randomly fluctuating structure. In the case of inelastic scattering, such as Neutron Spin Echo (NSE) [4], the intermediate scattering function is measured experimentally and usually reported in normalised way as I(q,τ)/I(q,0).

In addition to its relation to scattering, the covariance also carries important structural and dynamical information. This is notably the case for the specific surface area $a_{V}$ and the timely crossing rate $n_{t}$, which are obtained from $C_{11}\left(r,\tau \right)$ at small *r* and τ, namely

(5) $$C_{11}\left(r,\tau \right)≃\phi_{1}-\frac{a_{V}}{4}r-\frac{n_{t}}{2}\tau +\ldots$$

which applies to any two-phase structure. The specific surface area $a_{V}$ is defined as the area of the solid/pore interface per unit volume of the material, and its general relation to the covariance was first derived by Debye [27]. The small-*r* asymptotic behaviour in Equation (5) converts to the following large-*q* asymptotic scattering [2,3,28]

(6) $$I\left(q,0\right)≃2\pi \frac{a_{V}}{q^{4}}$$

which is referred to as Porod’s law. The timely crossing rate $n_{t}$ is defined as the average number of times a fixed point in space x is crossed by a moving interface of the structure during a time *t*. The value of $n_{t}$ controls the shape of the intermediate scattering function for large values of *q* and asymptotically small values of τ [19].

### 2.2. The Hierarchical Model

#### 2.2.1. Static Properties: Elastic Scattering

**Figure 1:**
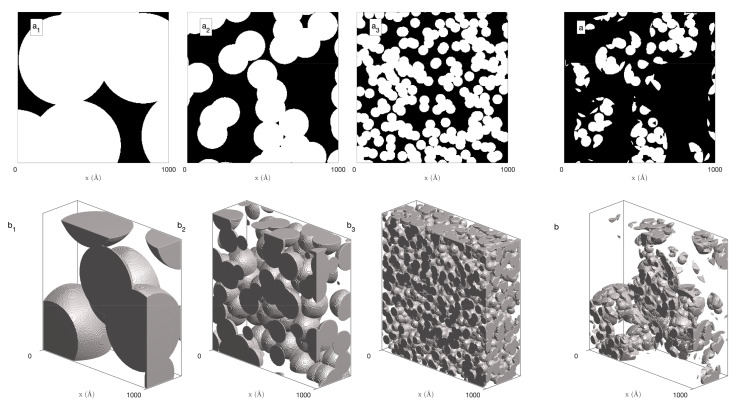
Hierarchical multi-scale model built as the intersection of Boolean models of disks (**top**) or spheres (**bottom**) with radii 300 Å (**a**${}_{1}$,**b**${}_{1}$), 100 Å (**a**${}_{2}$,**b**${}_{2}$) and 33 Å (**a**${}_{3}$,**b**${}_{3}$). The density of spheres at each level corresponds to a solid fraction 0.53, yielding an overall solid fraction $\phi_{1}=0.15$ in the hierarchical structure (**a**,**b**).

A general strategy for modelling multi-scale hierarchical structures consists in intersecting a variety of one-scale structures [16,18,29,30,31]. Our present approach is sketched in Figure 1, where the 2D case is meant only for visual clarity. We focus for now on the static case, and we generalize the results to time-dependent structures in Section 2.2.2.

Each level in the hierarchy of Figure 1 is a Boolean model, consisting of penetrable spheres with identical radii *R* and centres distributed in space according to a homogeneous Poisson point process with density θ [7,8,9,10,17,32]. The two parameters of the model—sphere radius *R* and density θ—control all structural characteristics. In particular, the pore fraction is calculated as

(7) $$\phi_{0}=\exp\left[-\eta \right]$$

with $\eta =\theta \left(4/3\right)\pi R^{3}$. The covariance of the pore space is calculated as

(8) $$C_{00}\left(r\right)=\exp\left[-2\eta +\eta K\left(r\right)\right]$$

where K(r) is the geometrical covariogram of the sphere with radius *R*, corresponding to the intersection volume of two identical spheres translated by a distance *r*, normalized by the sphere volume, namely

(9) $$K\left(r\right)={\left(1-\frac{r}{2R}\right)}^{2}\left(1+\frac{r}{4R}\right)$$

for r<2R and K=0 for larger distances. The solid fraction $\phi_{1}$ and covariance $C_{11}\left(r\right)$ are obtained as

(10) $$\phi_{1}=1-\phi_{0}$$

and

(11) $$C_{11}\left(r\right)=C_{00}\left(r\right)+1-2\phi_{0}$$

which are valid for any two-phase structures. Finally, the specific surface area of the Boolean model is calculated as

(12) $$a_{V}=\phi_{0}\times \theta 4\pi R^{2}$$

which results from Equation (5) through an expansion of Equation (8).

In the case of Figure 1, three hierarchical levels are considered with radii R=300 Å, 100 Å, and 33 Å, and their density θ was chosen such that the solid fraction $\phi_{1}$ of each level is 0.53. The overall structure is then obtained by intersecting all levels (Figure 1a,b). Formally, the indicator function of the hierarchical structure is written as the following product:

(13) $$I\left(x\right)=\prod_{i=1}^{N}I^{\left(i\right)}\left(x\right)$$

where each factor is the indicator function of a Boolean model of spheres with radius $R_{i}$ and density $\theta_{i}$. Most quantities of interest to the present study result from Equation (13) and from the statistical independence of each factor $I^{\left(i\right)}\left(x\right)$ from the others. In particular, the solid fraction of the hierarchical model is also a product

(14) $$\phi_{1}=\prod_{i=1}^{N}\phi_{1}^{\left(i\right)}$$

as a consequence of Equation (2). In this equation, $\phi_{1}^{\left(i\right)}$ is the solid fraction of the *i*th Boolean model. As discussed by Savary and Jeulin [29,33], intersecting independent Boolean models yield a structure with a low percolation threshold. Indeed, if the solid fraction is above the percolation threshold at each level ($\phi_{1}^{\left(i\right)}>0.31$ for a 3D model [10]) and if the radii $R_{i}$ are sufficiently different from one level to the next, then the hierarchical structure percolates as well. In the case of Figure 1—with $\phi_{1}^{\left(i\right)}≃0.53$, well above the 3D percolating threshold—the structure is connected although the solid fraction is as low as $\phi_{1}≃0.52^{3}≃0.15$. In principle, the percolating threshold can be made arbitrarily small by increasing the number *N* of hierarchical levels.

In addition to being hierarchical, the structure of many aerogels is self-similar with fractal dimension close to d=2 [22,34,35,36]. The model in Figure 1 can be made self-similar using Boolean models having identical volume fractions, $\phi_{1}^{\left(i\right)}=\varphi_{1}$ for all *i*’s, and radii in geometric progression, namely

(15) $$R_{i}=R_{1}\beta^{i-1}$$

where $R_{1}$ is the radius of the largest spheres and β<1. The corresponding fractal dimension is obtained by noting that the total solid volume in a region with size *L* is expected to scale like $L^{d}$ [37,38]. By construction, that region contains geometrically similar sub-regions that are β times smaller and occupy a fraction $\varphi_{1}$ of the original volume. The mass in each sub-region is $\beta^{d}$ smaller. On the other hand, the number of sub-regions is $\varphi_{1}\times \beta^{-3}$, where the exponent 3 accounts for the three-dimensionality of space. Self-similarity then demands $\varphi_{1}\beta^{d-3}=1$. This provides the fractal dimension

(16) $$d=3-\frac{\ln(\varphi_{1})}{\ln(\beta )}$$

as a function of model parameters β and $\varphi_{1}$. Strict self-similarity requires infinitely many hierarchical levels, which is never encountered in practice. Using a finite number of levels, *N*, the smallest sphere in the hierarchy has radius $R_{c}=R_{1}\beta^{N-1}$, which plays the role of a lower cutoff size for the construction.

In order to build an intuitive understanding of the model parameters, it is interesting to use Equation (16) to relate the solid fraction of the hierarchical structure $\phi_{1}=\varphi_{1}^{N}$ to the fractal dimension *d* as follows:

(17) $$\phi_{1}={\left(\frac{R_{c}}{R_{1}}\beta \right)}^{3-d}$$

**Figure 2:**
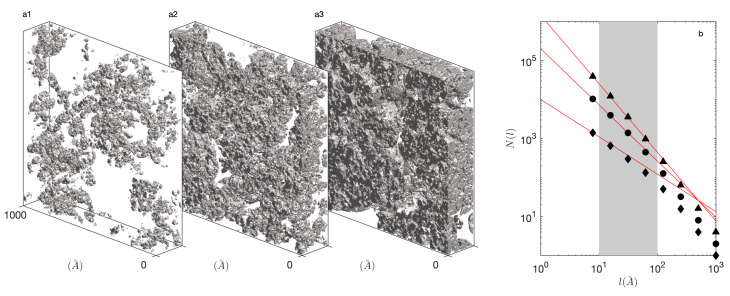
Realisations of a self-similar hierarchical model with $R_{1}=100$ Å, $R_{c}=10$ Å, N=5 intermediate structural levels, and with fractal dimensions d=2 (**a**${}_{1}$), d=2.4 (**a**${}_{2}$) and d=2.8 (**a**${}_{3}$). The box-counting determination of the fractal dimension from two-dimensional slices in the realizations is shown in (**b**) for d=2 (⧫), d=2.4 (•) and d=2.8 (▴). The grey area highlights the upper and lower cutoff sizes, and the expected slopes of the two-dimensional box-counting, $N\left(l\right)∼l^{1-d}$, are shown in red.

Here, $R_{c}/R_{1}$ is the scale over which the hierarchy is observed, which seldom exceeds one decade in natural systems [39]. A typical value is therefore $R_{c}/R_{1}≃1/10$. Moreover, if we assume an underlying bottom-up aggregation process, whereby two particles aggregate to form a cluster, two of which aggregate to form a larger cluster, etc. it is natural to assume a size ratio β≃1/2. With these orders of magnitude, Equation (17) predicts a density $\phi_{1}≃0.05$ for d=2, $\phi_{1}≃0.2$ for d=2.5, and $\phi_{1}≃0.5$ for d=2.8. These values are in reasonable agreement with those encountered in actual gels [22,34,35,36]. Corresponding structures are illustrated in Figure 2.

To confirm the self-similar nature of the generated structures, a box-counting analysis of the realisations is reported in Figure 2b. Accordingly, the simulated volume is decomposed into boxes with side-length *l*, and N(l) is the number of boxes that contain some solid. In the case of a fractal structure, a power-law dependence is expected of the type $N\left(l\right)≃l^{-d}$, where *d* is the fractal dimension. In the case of Figure 2, the box-counting was performed on two-dimensional slices in the realisation so that an exponent 1−d is observed [37]. As expected, the power law is observed only for box sizes intermediate between the two cutoff radii $R_{c}$ and $R_{1}$.

**Figure 3:**
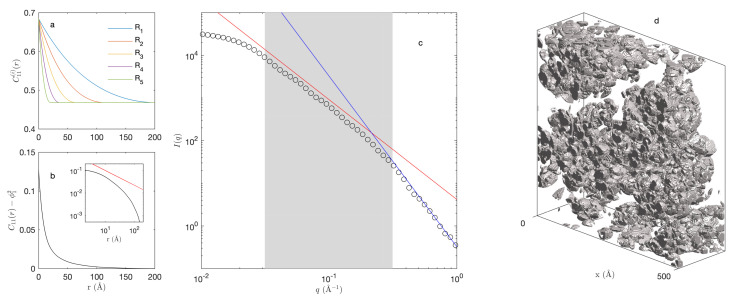
Covariance and elastic small-angle scattering pattern of the hierarchical model: (**a**) covariances of the Boolean models from $R_{1}=100$ Å to $R_{c}=10$ Å with N=5 intermediate levels, and fractal dimension d=2.3, (**b**) covariance of the resulting hierarchical structure, (**c**) scattered intensity of the hierarchical model (∘), and (**d**) realisation of the model. The inset in (**b**) plots the covariance on double logarithmic scales, together with the power law $r^{d-3}$ (red). The solid lines in (**c**) are Porod’s law $I=2\pi a_{V}q^{-4}$ (blue) and fractal scattering $I∼q^{-d}$ (red) with the shaded area highlighting the limits of the fractal regime from $q≃\pi /R_{1}$ to $\pi /R_{c}$.

The statistical independence of the various structural levels in the model enables one to evaluate the covariance of the hierarchical structure as the following product:

(18) $$C_{11}\left(r\right)=\prod_{i=1}^{N}C_{11}^{\left(i\right)}\left(r\right)$$

which is a consequence of Equation (3). In this equation, $C_{11}^{\left(i\right)}\left(r\right)$ is the covariance of the *i*th Boolean model, calculated through Equation (8). The various contributions to the covariance of the fractal model are illustrated in Figure 3, together with the elastic scattering pattern I(q,0), calculated through Equation (4). The scattering pattern exhibits a clear fractal scattering regime $I≃q^{-d}$ in a scattering vector range between $q=\pi /R_{1}$ and $\pi /R_{c}$ (grey area in Figure 3b). In principle, self-similarity is expected to manifest itself also in real space through a covariance of the type [37]:

(19) $$C_{11}\left(r\right)-\phi_{1}^{2}∼r^{d-3}$$

whose trend is barely visible in the inset Figure 3b. Self-similarity is much clearer in the scattering pattern, which makes small-angle scattering an ideal method to investigate it experimentally.

For scattering vectors larger than approximately $q≃\pi /R_{c}$, the intensity I(q,0) displays Porod scattering proportional to the specific surface area $a_{V}$ and with distinctive exponent 4, as predicted by Equation (6). The specific surface area of the hierarchical model is obtained from the small-*r* expansion of the covariance as in Equation (5). From Equation (18), one obtains

(20) $$a_{V}=\phi_{1}\sum_{i=1}^{N}\frac{a_{V}^{\left(i\right)}}{\phi_{1}^{\left(i\right)}}$$

where $a_{V}^{\left(i\right)}$ is the the surface area of the *i*th structural level, calculated through Equation (12). The blue line in Figure 3c was obtained from the so-calculated surface area.

#### 2.2.2. Dynamic Properties: Inelastic Scattering

**Figure 4:**
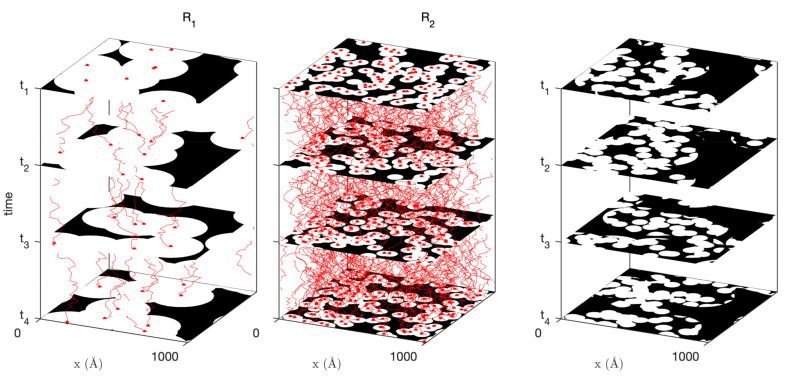
Two-dimensional illustration of the time-dependent generalization of the hierarchical model of Figure 1, whereby the centres of the disks at each sub-level (with radii $R_{1}$ and $R_{2}$) move according to some user-defined and possibility scale-dependent probabilistic rules. The hierarchical time-dependent model (right) is obtained as before by intersecting all sub-structures. The trajectories of the sphere centres are shown in red.

The model presented in Section 2.2.1 describes a static structure. In order to make the model susceptible to being used for inelastic neutron scattering data analysis, we now generalize it to make it time-dependent. The idea underlying the generalisation is sketched in Figure 4: it consists of letting the grains that make up the Boolean models move according to some user-defined rules.

In the context of stochastic models, it is sufficient to probabilistically describe the motion of the centers of the grains. This is done by considering the jump probability distribution $f_{\tau }\left(j\right)$ such that $f_{\tau }\left(j\right)dV$ is the conditional probability for a center initially at position x to be found in an infinitesimal volume dV containing x+j at time τ. A natural case to consider is when the grains diffuse with diffusion coefficient *D*, which corresponds to the following jump distribution [19,40]:

(21) $$f_{\tau }\left(j\right)=\frac{1}{{\left(4\pi D\tau \right)}^{3/2}}\exp\left[-\frac{{\left|j\right|}^{2}}{4D\tau }\right]$$

This diffusive model is the one shown in Figure 4. A qualitatively different dynamics is obtained by letting the grains move at constant velocity *c* along randomly oriented straight lines. This corresponds to a ballistic motion, characterized by [19]

(22) $$f_{\tau }\left(j\right)=\frac{1}{{4\pi |j|}^{2}}\delta \left[|j|-c\tau \right]$$

where δ[] is Dirac’s delta function, and the denominator accounts for the normalisation of $f_{\tau }\left(j\right)$.

The inelastic scattering from a dynamical system as in Figure 4 depends on its covariance $C_{11}\left(r,\tau \right)$ for finite values of τ. As this question does not seem to have been investigated so far, we calculate here a general expression for the time-dependent covariance of the Boolean model with moving grains. For readers familiar with Boolean models, the final result in Equation (28) can be obtained by simply noting that $C_{00}\left(r,\tau \right)$ is mathematically equivalent to the porosity $\phi_{0}$ of a static Boolean model, in which the primary grain is the intersection of two grains of the original model shifted by j−r, with j having density distribution $f_{\tau }\left(j\right)$. Other readers may find the following self-contained derivation useful, although it is limited to spherical grains.

**Figure 5:**
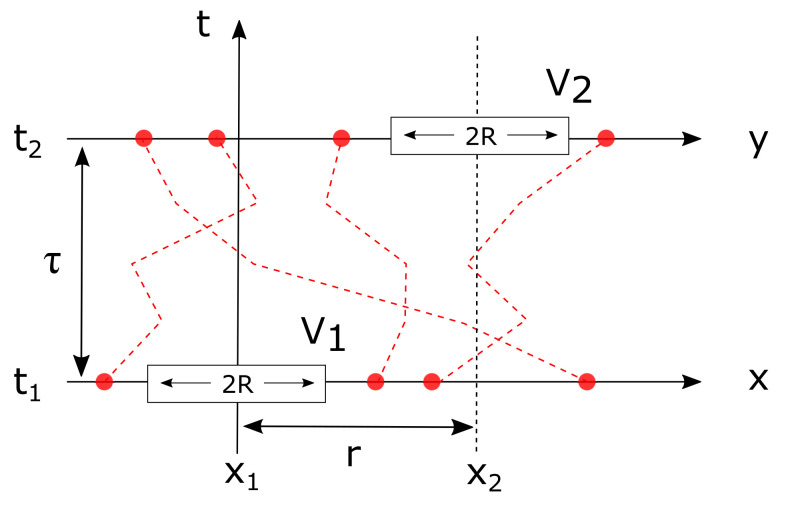
Calculation of the covariance of the time-dependent Boolean model (one-dimensional, sketch). The centers of the moving grains are shown in red. The covariance $C_{00}\left(r,\tau \right)$ is the probability that the volumes $V_{1}$ and $V_{2}$ at distance *r* from one another do not contain any grain center at times $t_{1}$ and $t_{2}=t_{1}+\tau$.

Consider the covariance of the pores $C_{00}\left(r,\tau \right)$, corresponding to the joint probability that point $x_{1}$ is in the pores at time $t_{1}$ and point $x_{2}=x_{1}+r$ is in the pores are time $t_{2}=t_{1}+\tau$. The situation is sketched in Figure 5, and the calculation of $C_{00}\left(r,\tau \right)$ is based on the observation that a given point, say $x_{1}$, is in the pores at time $t_{1}$ if all grain centers are at distance larger than *R*. This is equivalent to defining a spherical volume centred on $x_{1}$ with radius *R* from which all grain centers are excluded. In the 1D sketch of Figure 5, these are shown as segments $V_{1}$ and $V_{2}$, each of which has length 2R. The actual grains, of which the centers are shown in red, are not shown in the figure.

Consider specifically the case of volume $V_{2}$ at times $t_{2}$, and assume that the grains are uniformly distributed at time $t_{1}$ with density θ. For the calculation, we discretize space an infinite number of elementary volumes with volume $d^{3}x$ centred on points $x_{i}$. The probability that $V_{2}$ is empty of any grain center at time $t_{2}$ is calculated as

(23) $$\mathrm{Prob}\left\{V_{2}\;\mathrm{empty}\right\}=\prod_{i}\left[\left(1-\theta d^{3}x\right)+\theta d^{3}x\int_{V_{2}^{C}}d^{3}y\;f_{\tau }\left(y-x_{i}\right)\right]$$

where the product is on all elementary volumes of space at time $t_{1}$, each of which has probability $1-\theta d^{3}x$ to be empty and probability $\theta d^{3}x$ to contain a seed. The second term in the product is proportional to an integral over $V_{2}^{C}$ (the complementary of $V_{2}$), which is the conditional probability that a grain center be outside $V_{2}$ at time $t_{2}$ given that it is at point $x_{i}$ at time $t_{1}$. In the limit of $\theta d^{3}x\to 0$, this becomes

(24) $$\ln\left[\mathrm{Prob}\left\{V_{2}\;\mathrm{empty}\right\}\right]=-\theta \int_{R^{3}}d^{3}x\int_{V_{2}}d^{3}y\;f_{\tau }\left(y-x\right)$$

As the orders of the integrals can be inverted and $f_{\tau }\left(j\right)$ is normalized to one, this corresponds to the classical result

(25) $$\mathrm{Prob}\left\{V\;\mathrm{empty}\right\}=\exp\left[-\theta V\right]$$

which is identical to Equation (7) because the excluded volume *V* is geometrically identical to the sphere.

With the same line of reasoning, the covariance $C_{00}\left(r,\tau \right)$ is calculated from the conditional probability that $V_{2}$ be empty at time $t_{2}$ given that $V_{1}$ is empty at time $t_{1}$, namely

(26) $$C_{00}\left(r,\tau \right)=\mathrm{Prob}\left\{V_{2}\;\mathrm{empty}\mathrm{at}\;t_{2}|V_{1}\;\mathrm{empty}\mathrm{at}\;t_{1}\right\}\times \mathrm{Prob}\left\{V_{1}\;\mathrm{empty}\right\}$$

The conditional probability is obtained from Equation (24) by replacing the integral on $R^{3}$ by an integral over $V_{1}^{C}$ (the complementary of $V_{1}$). This can eventually be written as

(27) $$\ln\left[\mathrm{Prob}\left\{V_{2}\;\mathrm{empty}|V_{1}\;\mathrm{empty}\right\}\right]=\ln\left[\mathrm{Prob}\left\{V\;\mathrm{empty}\right\}\right]+\theta \int_{V_{1}}d^{3}x\int_{V_{2}}d^{3}y\;f_{\tau }\left(y-x\right)$$

where the integral is on $V_{1}$ and $V_{2}$, not their complementary. Using Equation (25), the following expression is finally obtained for the time-dependent covariance

(28) $$C_{00}\left(r,\tau \right)=\exp\left[-2\eta +\eta K(r,\tau ))\right]$$

where the double integral that appears in Equation (27) was written as V×K(r,τ). The latter can be expressed as follows:

(29) $$K\left(r,\tau \right)=\int d^{3}j\;K\left(r-j\right)f_{\tau }\left(j\right)$$

as the convolution of the jump probability distribution $f_{\tau }\left(j\right)$ with the geometrical covariogram K(r) of the grain.

In the important case where the grains and their displacements are statistically isotropic, both the covariogram and the jump probabilities are radial functions, so that the convolution in Equation (29) can be written as the following double integral

(30) $$K\left(r,\tau \right)=2\pi \int_{0}^{\infty }\rho^{2}d\rho \int_{-1}^{+1}d\mu \;f_{\tau }\left(\sqrt{r^{2}+\rho^{2}-2\rho r\mu }\right)K\left(\rho \right)$$

**Figure 6:**
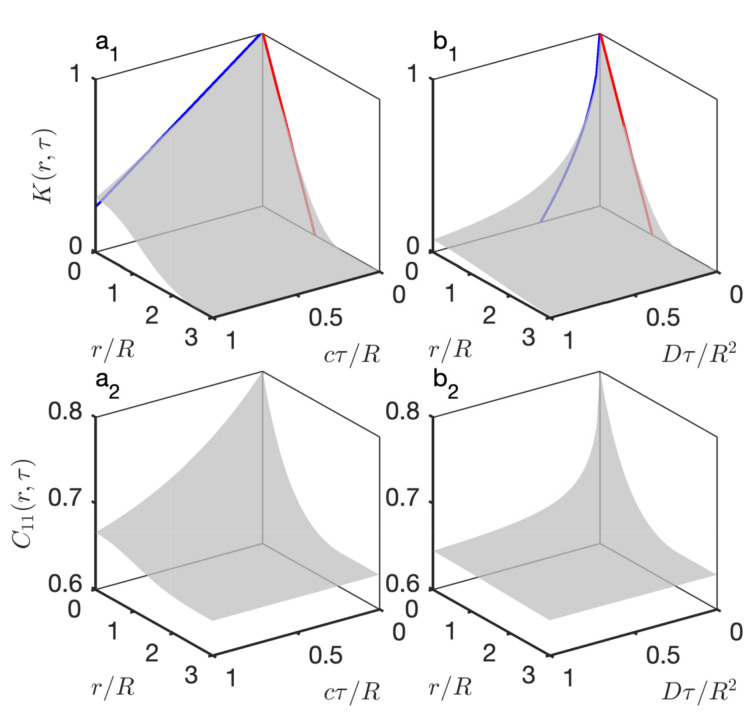
Time-dependent covariogram K(r,τ) of spherical grains with ballistic (**a**${}_{1}$) and diffusive (**b**${}_{1}$) movements, plotted against dimensionless distance r/R and times cτ/R and $D\tau /R^{2}$. The red and blue lines are the asymptotic relations at the origin. The covariances of the corresponding Boolean model, assuming $\phi_{1}=0.8$ are shown in (**a**${}_{2}$,**b**${}_{2}$).

In order to use the models in fitting procedures, it proved convenient to derive complete analytical expressions for K(r,τ) that can be numerically evaluated very fast. They are provided in the Supporting Information for the cases of ballistic and diffusive spheres, in Equations (S-6) and (S-26), respectively. The so-calculated covariograms and corresponding covariances of the Boolean model (evaluated through Equation (28) assuming $\phi_{1}=0.8$) are shown in Figure 6, for both the ballistic and diffusive cases.

The shape of the time-dependent covariogram K(0,τ) for vanishingly small times is qualitatively different for the ballistic and the diffusive motions, as it is linear in Figure 6a_1_ and singular in Figure 6b_1_. This can be understood by noting that, for any convex grain with volume *V* and surface area *A*, the geometrical covariogram obeys K(r)≃1−Ar/(4V)+… for asymptotically small distances (red lines in Figure 6). Building on that observation, Equation (30) provides the following general relation for the time-dependent covariogram:

(31) $$K\left(0,\tau \right)≃1-\frac{A}{4V}{\langle j\rangle }_{\tau }+\ldots$$

where ${\langle j\rangle }_{\tau }$ is the average length of the jump over time interval τ, calculated as

(32) $${\langle j\rangle }_{\tau }=4\pi \int_{0}^{\infty }f_{\tau }\left(\rho \right)\rho^{3}d\rho$$

In the case of the ballistic model and spherical grains, this yields

(33) $$K\left(0,\tau \right)=1-\frac{3}{4}\frac{c\tau }{R}+\ldots$$

which is plotted as a blue line in Figure 6a_1_. In the case of the diffusive model, the asymptotic relation is

(34) $$K\left(0,\tau \right)=1-\frac{3}{\sqrt{\pi }}\sqrt{\frac{D\tau }{R^{2}}}+\ldots$$

which has an infinite slope of for τ→0 (blue line in Figure 6b_1_). The shape of K(0,τ) controls the shape of the covariance at the origin, which in turn controls the crossing rate $n_{t}$ through Equation (5). The nonlinear behaviour of $C_{00}\left(0,\tau \right)$ for the diffusive model testifies to an infinite crossing rate, which is often a signature of thermal fluctuations [19]. By contrast, the crossing rate is finite in the case of the ballistic model, and given by

(35) $$n_{t}=\frac{c}{2}a_{V}$$

with the specific surface area $a_{V}$ given by Equation (12).

**Figure 7:**
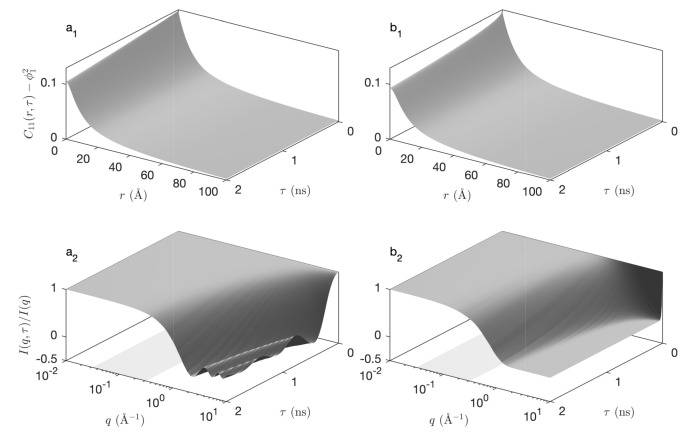
Time-dependent covariance $C_{11}\left(r,\tau \right)-\phi_{1}^{2}$ (**a**${}_{1}$,**b**${}_{1}$) of the fractal structure of Figure 3 and corresponding intermediate scattering function I(q,τ)/I(q) (**a**${}_{2}$,**b**${}_{2}$), assuming ballistic dynamics (**a**${}_{1}$,**a**${}_{2}$, with c=1 Å/ns) and diffusive dynamics (**b**${}_{1}$,**b**${}_{2}$, with D=1 Å${}^{2}$/ns). The flat grey areas in (**a**${}_{2}$,**b**${}_{2}$) highlight the *q* range over which the elastic scattering pattern exhibits a fractal power law (same as Figure 3).

For calculating the time-dependent covariance of the hierarchical model, Equation (18) applies unchanged provided the static covariance $C_{11}^{\left(i\right)}\left(r\right)$ is replaced by the time-dependent covariance of the Boolean model $C_{11}^{\left(i\right)}\left(r,\tau \right)$. The latter is obtained by evaluating Equation (28) and using the general result in Equation (11) to convert the pore covariance $C_{00}\left(r,\tau \right)$ to the solid covariance $C_{11}\left(r,\tau \right)$. The so-obtained covariances are plotted in Figure 7a_1_,b_1_ for ballistic and diffusive dynamics (assuming c=1 Å/ns or D=1 Å${}^{2}$/ns for all levels in the hierarchy). The quantity that is measured experimentally is not directly $C_{11}\left(r,\tau \right)$ but the intermediate scattering functions, which are obtained by a Fourier transform through Equation (4). The latter are plotted in Figure 7a_2_,b_2_.

The overall shape of the intermediate scattering function is the same for the ballistic and diffusive dynamics, with a plateau at I(q,τ)/I(q,0)≃1 for small *q* and τ, and a sharp transition towards for large *q* and τ. The distinctive differences between the intermediate scattering functions of the ballistic and diffusive dynamics are the presence of oscillations in the ballistic case, and the steepness of I(q,τ) for large *q* and small τ, which is infinite in the case of the diffusive model. The latter observation is a direct consequence of the qualitative difference between the time-dependent covariograms K(r,τ) already noticed when discussing Figure 6.

### 2.3. Discussion

**Figure 8:**
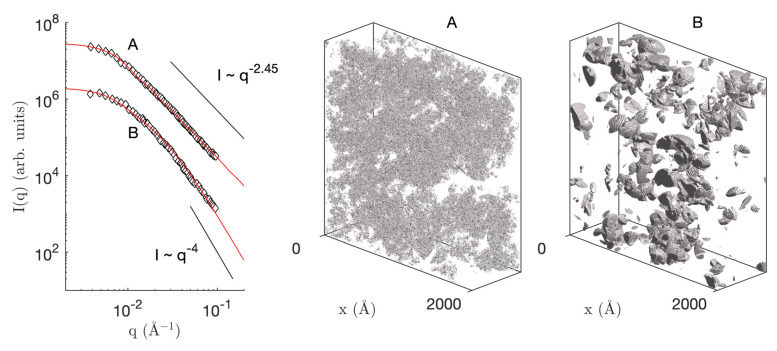
(**Left**) small-angle neutron scattering patterns of silica aerogels obtained by acid-catalyzed (**A**) and two-step acid-base (**B**) syntheses. The symbols are the data taken from Ref. [23] and the solid red lines are the best fits with the fractal model; (**Right**) corresponding realizations of the fitted model.

**Figure 9:**
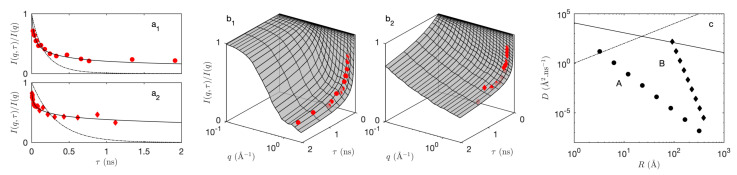
(**Left**) experimental intermediate scattering functions (symbols) measured at q=1.55 Å${}^{-1}$ on the acid-catalyzed (**a**${}_{1}$) and two-step acid-base catalyzed (**a**${}_{2}$) aerogels [23]. The dashed line is the best fit with a single diffusion coefficient *D* and the solid line is the best fit with an additional scaling exponent δ through Equation (37). (**Middle**) (**b**${}_{1}$,**b**${}_{2}$) complete intermediate scattering functions over a broader *q* range; (**Right**) (**c**) fitted scale-dependent diffusion coefficients on the acid (A) and two-step acid-base (B) aerogels. The Stokes–Einstein relation in air is shown as a solid line, and the relation $D=R^{2}/\mathrm{ns}$ is shown as a dashed line.

To illustrate the use of the models developed in the paper, we use them to analyze some of the elastic and inelastic neutron scattering data measured by Schaefer et al. on a variety of silica aerogels [23]. The small-angle neutron scattering (SANS) of aerogels obtained through acid-catalysis and two-step acid-base process are plotted in Figure 8 as I(q). The corresponding inelastic scattering data I(q,τ)/I(q) measured at q=1.55 Å${}^{-1}$ on a neutron spin-echo (NSE) instrument are reported in Figure 9a_1_,a_2_.

For the fitting of the SANS data, the fractal model was used, based on three adjustable parameters, namely: (i) the radius of the largest spheres $R_{1}$ in the hierarchy, corresponding to the length scale above which the material is homogeneous, (ii) the lower cutoff radius $R_{c}$, and (iii) the number *N* of intermediate hierarchical levels in between, assuming that their radii are in geometric progression between $R_{1}$ and $R_{c}$. The solid fraction $\phi_{1}$ is not adjusted as it is imposed by the known density of the aerogel, namely 0.16 and 0.15 g/cm^3^ for aerogels A and B, respectively [23]. Assuming 2.65 g/cm^3^ for the skeletal density of silica, the corresponding volume fractions are $\phi_{1}=0.06$ and 0.057.

The fitting procedure is described in detail in the Supporting Information, notably in Figures S1 and S2. For both aerogels, the fit leads to an upper radius around $R_{1}≃390$ Å. The main difference between the two materials is the lower cutoff $R_{c}$. In the case of aerogel A, the fractal power-law scattering extends beyond the upper *q* limit of the data and the fit converges to the lowest allowed value $R_{c}≃3$ Å. For aerogel B, a clear downward deviation towards Porod’s $I∼q^{-4}$ law is observed within the experimental *q* range and the fitted value is $R_{c}≃90$ Å. For both aerogels, good fits of the SANS data are obtained with about N≃10 levels in the hierarchical structure. The fractal dimension calculated from the fitted parameter through Equation (16) is d≃2.45 for aerogel A and d≃1.2 for aerogel B. The former value is in line with generally reported values for this type of material. In the case of aerogel B, the value of *d* is meaningless as the upper and lower cutoff lengths are too close to each other for any scale-invariance consideration to be relevant.

An interesting difference between the two aerogels is the size of the smallest objects, which are often thought of as the particles that have aggregated to form the structure. As a consequence of the intersection process through which the model is created (Figure 1), the smallest homogeneous lumps in the structure can be significantly smaller than $R_{c}$. An estimation of their size is provided by the equivalent diameter, $d_{eq}$, defined as the diameter of the sphere that has the same surface to volume ratio as the solid, namely

(36) $$d_{eq}=\frac{6\phi_{1}}{a_{V}}$$

Using Equation (20) to calculate the specific surface area, the fitted parameters convert to $d_{eq}≃2$ Å for aerogel A, and $d_{eq}≃30$ Å for aerogel B. These values point at almost molecular-sized building blocks in the acid-catalyzed aerogel and to nanometer-sized building blocks in the two-step acid-base aerogel, as expected [23,36].

In addition to the elastic scattering data—which offers a static snapshot of the aerogels structures—the inelastic neutron scattering data in Figure 9 provide us with invaluable information about their nanometer-scale motion over nanoseconds. A classical procedure to analyze inelastic neutron scattering patterns in aerogels consists of converting the data into a density of state as a function of the vibration frequency ω [23,41]. The latter can be interpreted in terms of different deformation mechanisms with phonons at low frequency and a progressive transition towards localised modes (so-called fractons) at higher frequency [42,43]. The structural insight obtained with such approach is only indirect, as it is difficult to relate the time frequency to a spatial scale. Moreover, the elastic and inelastic scattering are analyzed independently from one another, so that the dynamic information can only be expressed in general terms that are not specific to the structure.

An interesting aspect of the models developed in the paper is the possibility they offer to jointly analyze the elastic and inelastic scattering data within a single time-dependent structural model. For that purpose, the structural parameters identified from the elastic scattering data are complemented with dynamical parameters that are evaluated from the inelastic data. In that spirit, we first observe that the experimental intermediate scattering functions in Figure 9a_1_,a_2_ do not exhibit any oscillation, and have an infinite slope for τ→0. These two qualitative observations rule out the ballistic model (Figure 7a_2_) and hint at a diffusive dynamics (Figure 7b_2_) with infinite crossing rate $n_{t}$ [19].

As a first attempt to describe the inelastic scattering data of the aerogels in terms of diffusing aggregates, we considered the situation where all aggregates have the same diffusion coefficient *D*, irrespective of their size. This simple model—with structural parameters $R_{1}$, $R_{c}$ and *N* fixed by the SANS—is not able to account for the shape of the intermediate scattering function as illustrated with the dashed lines in Figure 9a_1_,a_2_. In order to account for the scale-dependent mobility of the aggregates, we assumed the following size-dependence of the diffusion coefficients

(37) $$D=D_{1}{\left(\frac{R_{1}}{R}\right)}^{\delta }$$

where $D_{1}$ is the diffusion coefficient of the largest aggregates for $R=R_{1}$, and δ is a the scaling exponent. This two-parameter model captures very nicely the inelastic scattering data (solid lines in Figure 9a_1_,a_2_). The so-obtained size-dependent diffusion coefficients of the two aerogels are plotted in Figure 9c.

As expected, the aggregates are found to have very little mobility at the scale of $R_{1}$, at which they are tightly connected to each other to form the solid network of the aerogel. It is important to notice that the distance travelled by a diffusing object scales with time like $\sqrt{D\tau }$. Over a duration τ=2 ns relevant to the inelastic scattering data and with a diffusion coefficient of the order of 10${}^{-6}$ Å${}^{2}$/ns, the deformation of the structure is found to be infinitesimal at the scale of $R_{1}$. Therefore, the assumption of a diffusive motion does not conflict with the elasticity of the aerogel at a macroscopic scale. When exploring the structure at smaller and smaller scales, from $R_{1}$ down to $R_{c}$, the connectivity of the aggregates is expected to become looser and looser as aerogels are known to possess dangling branches that do not contribute to the connectivity of the structure [44,45]. In the present analysis, this is manifested in the positive values of the scaling exponent δ and the steep slopes in Figure 9c. At the smallest scale, the diffusion coefficients of the acidic aerogel point to movement with a large amplitude, as the particles move over distances comparable to their size in τ=1 ns (dashed line in Figure 9c).

Additional insight is obtained by comparing the diffusion coefficients derived from inelastic scattering with those predicted by the Stokes–Einstein relation. The latter estimates the diffusion coefficient of a free-standing spherical nanoparticle with radius *R* as

(38) $$D=\frac{k_{B}T}{6\pi \eta R}$$

where $k_{B}$ is Boltzman’s constant, *T* is the temperature, and η is the viscosity of the surrounding fluid. The value calculated assuming the viscosity of air is plotted as a solid line in Figure 9. The diffusion coefficients of the aggregates in the aerogels are smaller than those of free-standing spheres with the same size, as expected. In the case of smallest aggregates in aerogel B, however, the two values are comparable, which proves that the structure is extremely poorly connected at that scale. By contrast, the smallest structures in the acid-catalyzed aerogel appear to be very well connected, as the diffusion coefficient is two orders of magnitude smaller than the Stokes–Einstein value. Such type of high connectivity at the molecular scale is indirectly supported by the observation of entropic elasticity in acid-catalyzed aerogels [46].

A striking difference between aerogels A and B is the value of the scaling exponent δ, corresponding to the slopes of the scaling laws in Figure 9c. For the acid aerogel, the exponent is found to be δ≃4. Such value could be explained with a scale-dependent viscosity following $\eta ≃R^{3}$, i.e., with an exponent comparable to those found in reticulated polymers [47]. For the two-step aerogel B, a much larger scaling exponent is found δ≃12, hinting at a qualitatively different type of network connectivity [48].

## 3. Conclusions

We have developed a general stochastic model to describe the time-dependent structure of hierarchical nanostructured materials undergoing thermal fluctuations. The model contains self-similar (fractal) structures as a particular case, but it is not limited to them. The time-dependence is described through a generalisation of the Boolean model of penetrable spheres, whereby the spheres are allowed to move in space either diffusively or ballistically. Analytical expressions are derived for the space- and time-dependent covariance (van-Hove correlation function), which makes the model practical to analyze and fit elastic and inelastic neutron scattering data.

The use of the model was illustrated by re-analysing neutron scattering data measured on two silica aerogels, synthesised in acidic conditions or via a two-step acid-base process [23]. All the available small-angle neutron scattering (SANS) and neutron-spin echo (NSE) data could be described quantitatively with a single model. The model describes the structures of the aerogels and their thermal fluctuations in terms of hierarchical aggregates with sizes ranging from 400 Å down to molecular dimensions with scale-dependent diffusion coefficients. In the acidic aerogels, the structures are found to be well connected down to almost molecular dimensions. In the two-step acid-base aerogels, the smallest objects are nanometer-sized, and their mobility is comparable to that of free-standing particles, which points at very poor connectivity.

## Supplementary Materials

The following are available online at https://www.mdpi.com/article/10.3390/gels8040236/s1: analytical expressions for the time-dependent covariogram of spherical particles K(r,τ) for both ballistic and diffusive dynamics and details about the SANS fitting procedure.

## References

[1] Narayanan T., Wacklin H., Konovalov O., Lund R. (2017). Recent applications of synchrotron radiation and neutrons in the study of soft matter. Crystallogr. Rev..

[2] Glatter O., Kratky O. (1982). Small Angle X-ray Scattering.

[3] Sivia D.S. (2011). Elementary Scattering Theory.

[4] Holderer O., Zolnierczuk P., Pasini S., Stingaciu L., Monkenbusch M. (2019). A better view through new glasses: Developments at the Jülich neutron spin echo spectrometers. Phys. B Condens. Matter.

[5] Squires G.L. (2012). Introduction to the Theory of Thermal Neutron Scattering.

[6] Gommes C.J., Jiao Y., Torquato S. (2012). Microstructural degeneracy associated with a two-point correlation function and its information content. Phys. Rev. E.

[7] Matheron G. (1967). Éléments pour une Théorie des Milieux Poreux.

[8] Torquato S. (2002). Random Heterogeneous Materials.

[9] Lantuéjoul C. (2002). Geostatistical Simulations.

[10] Jeulin D. (2021). Morphological Models of Random Structures.

[11] Sonntag U., Stoyan D., Hermann H. (1981). Random set models in the interpretation of small-angle scattering data. Phys. Status Solidi.

[12] Berk N. (1987). Scattering properties of a model bicontinuous structure with a well defined length scale. Phys. Rev. Lett..

[13] Teubner M. (1991). Level Surfaces of Gaussian Random Fields and Microemulsions. Europhys. Lett..

[14] Chen S., Lee D., Kimishima K., Jinnai H., Hashimoto T. (1996). Measurement of the Gaussian curvature of the surfactant film in an isometric bicontinuous one-phase microemulsion. Phys. Rev. E.

[15] Roberts A.P. (1997). Morphology and thermal conductivity of model organic aerogels. Phys. Rev. E.

[16] Gommes C.J., Roberts A.P. (2008). Structure development of resorcinol-formaldehyde gels: Microphase separation or colloid aggregation. Phys. Rev. E.

[17] Gille W. (2011). Scattering properties and structure functions of Boolean models. Comput. Struct..

[18] Gommes C.J. (2018). Stochastic models of disordered mesoporous materials for small-angle scattering analysis and more. Microporous Mesoporous Mater..

[19] Gommes C.J., Zorn R., Jaksch S., Frielinghaus H., Holderer O. (2021). Inelastic neutron scattering analysis with time-dependent Gaussian-field models. J. Chem. Phys..

[20] Soler-Illia G., Sanchez C., Lebeau B., Patarin J. (2002). Chemical strategies to design textured materials: From microporous and mesoporous oxides to nanonetworks and hierarchical structures. Chem. Rev..

[21] Fratzl P., Weinkamer R. (2007). Nature’s hierarchical materials. Prog. Mater. Sci..

[22] Woignier T., Primera J., Alaoui A., Dieudonne P., Duffours L., Beurroies I., Calas-Etienne S., Despestis F., Faivre A., Etienne P. (2021). Fractal Structure in Silica and Composites Aerogels. Gels.

[23] Schaefer D.W., Brinker C.J., Richter D., Farago B., Frick B. (1990). Dynamics of weakly connected solids: Silica aerogels. Phys. Rev. Lett..

[24] Lantuejoul C. (1991). Ergodicity and integral range. J. Microsc..

[25] Van Hove L. (1954). Correlations in Space and Time and Born Approximation Scattering in Systems of Interacting Particles. Phys. Rev..

[26] Guinier A., Fournet G. (1955). Small-Angle Scattering of X-rays.

[27] Debye P., Anderson H., Brumberger H. (1957). Scattering by an inhomogeneous solid. II. the correlation function and its application. J. Appl. Phys..

[28] Ciccariello S., Goodisman J., Brumberger H. (1988). On the Porod law. J. Appl. Crystallogr..

[29] Jeulin D. (2012). Morphology and effective properties of multi-scale random sets: A review. C. R. Méc..

[30] Gommes C.J., Prieto G., Zecevic J., Vanhalle M., Goderis B., DeJong K.P., DeJongh P.E. (2015). Mesoscale Characterization of Nanoparticles Distribution Using X-ray Scattering. Angew. Chem.-Int. Ed..

[31] Gommes C.J., Prieto G., De Jongh P.E. (2016). Small-angle scattering analysis of empty or loaded hierarchical porous materials. J. Phys. Chem. C.

[32] Serra J. (1982). Image Analysis and Mathematical Morphology.

[33] Savary L., Jeulin D., Thorel A. (1999). Morphological analysis of carbon-polymer composite materials from thick sections. Acta Stereol..

[34] Schaefer D.W., Keefer K.D. (1984). Fractal geometry of silica condensation polymers. Phys. Rev. Lett..

[35] Vacher R., Woignier T., Pelous J., Courtens E. (1988). Structure and self-similarity of silica aerogels. Phys. Rev. B.

[36] Brinker C.J., Scherer G.W. (1990). Sol-Gel Science, the Physics and Chemistry of Sol-Gel Processing.

[37] Vicsek T. (1992). Fractal Growth Phenomena.

[38] Gouyet J.F. (1996). Phyisc and Fractal Structures.

[39] Malcai O., Lidar D.A., Biham O., Avnir D. (1997). Scaling range and cutoffs in empirical fractals. Phys. Rev. E.

[40] Berg H. (1993). Random Walks in Biology.

[41] Vacher R., Courtens E., Coddens G., Heidemann A., Tsujimi Y., Pelous J., Foret M. (1990). Crossovers in the density of states of fractal silica aerogels. Phy. Rev. Lett..

[42] Alexander S., Orbach R. (1982). Density fo states of fractals: Fractons. J. Phys. Lett..

[43] Amir A., Krich J.J., Vitelli V., Oreg Y., Imry Y. (2013). Emergent Percolation Length and Localization in Random Elastic Networks. Phys. Rev. X.

[44] Ma H., Jullien R., Scherer G.W. (2002). Dangling bond deflection model: Growth of gel network with loop structure. Phys. Rev. E.

[45] Danielsen S.P.O., Beech H.K., Wang S., El-Zaatari B.M., Wang X., Sapir L., Ouchi T., Wang Z., Johnson P.N., Hu Y. (2021). Molecular Characterization of Polymer Networks. Chem. Rev..

[46] Daughton D.R., MacDonald J., Mulders N. (2003). Acoustic properties of silica aerogels from 400 mK to 400 K. Phys. B Condens. Matter.

[47] Grosberg A.Y., Joanny J., Srinin W., Rabin Y. (2016). Scale-Dependent Viscosity in Polymer Fluids. J. Phys. Chem. B.

[48] Anglaret E., Pelous J., Hrubesh L.H. (1995). Structural changes and elastic properties in aerogels investigated by Brillouin scattering. J. Non-Cryst. Solids.

