# Supplementary material for: A Time-Dependent Hierarchical Model for Elastic and Inelastic Scattering Data Analysis of Aerogels and Similar Soft Materials
Source: Gels. 2022 Apr 12;8(4):236. doi: 10.3390/gels8040236 (PMC9025713; doi:10.3390/gels8040236)
Supplement: Supplementary file 1 [file gels-08-00236-s001.zip › gels-1611270-supplementary.pdf]

gels

MDPI

Article

A Time-Dependent Hierarchical Model for Elastic and Inelastic Scattering Data Analysis of Aerogels and Similar Soft Materials

Supplementary Material

Cedric J. Gommes

Department of Chemical Engineering, University of Liège B6C, Allée du Six Août 3, B-4000 Liège, Belgium; cedric.gommes@uliege.be

check for updates

Citation: Gommes, C.J. A Time-Dependent Hierarchical Model for Elastic and Inelastic Scattering Data Analysis of Aerogels and Similar Soft Materials. *Gels* **2022**, *8*, 236. https://doi.org/10.3390/gels8040236

Academic Editor: Ameya Rege

Received: 9 February 2022

Accepted: 1 April 2022

Published: 12 April 2022

Publisher's Note: MDPI stays neutral with regard to jurisdictional claims in published maps and institutional affiliations.

Copyright: © 2022 by the author. Licensee MDPI, Basel, Switzerland. This article is an open access article distributed under the terms and conditions of the Creative Commons Attribution (CC BY) license (https://creativecommons.org/licenses/by/4.0/).

We here derive analytical expressions for the time-dependent geometrical covariogram $K(r,\tau)$ of spherical particles undergoing either ballistic or diffusive motion. These expressions are useful for the fast numerical calculation of $K(r,\tau)$ and the least-square fitting of NSE data presented in the main text. We also provide details about the numerical procedure used in the main text for the fitting of the SANS data with the fractal model.

1. Analytical expressions for the time-dependent covariogram $K(r,\tau)$

The general relation in Equation (29) of the main text expresses that the time-dependent covariogram is the convolution of the geometrical covariogram with the displacement law, namely

$$K(r,\tau) = \int \mathrm{d}^3\mathbf{j}\; K(\mathbf{r}-\mathbf{j}) f_\tau(\mathbf{j}) \tag{S-1}$$

In the case where the motion of the grains is statistically isotropic, the function $f_\tau(\mathbf{j})$ depends only on the modulus $|\mathbf{j}|$. If additionally the grains are also isotropic, the the three-dimensional convolution in Equation (S-1) reduces to a two-dimensional integral, which can be easily evaluated numerically. Using spherical coordinates centred on $\mathbf{j}=0$ and with azimutal angle θ measured against the direction of $\mathbf{r}$, the convolution in Equation (S-1) can be written as

$$K(r,\tau) = 2\pi \int_0^\infty \rho^2 \mathrm{d}\rho \int_{-1}^{+1} \mathrm{d}\mu\; K(\sqrt{r^2+\rho^2-2\rho r\mu}) f_\tau(\rho) \tag{S-2}$$

where $\mu = \cos(\theta)$. From the commutativity of the convolution, this can be calculated equivalently as

$$K(r,\tau) = 2\pi \int_0^\infty \rho^2 \mathrm{d}\rho \int_{-1}^{+1} \mathrm{d}\mu\; f_\tau(\sqrt{r^2+\rho^2-2\rho r\mu}) K(\rho) \tag{S-3}$$

In the particular case of spherical grains the latter expression is easier to evaluate numerically, as the integral on ρ can be limited to the finite support of $K(r)$, namely from 0 to $2R$.

1.1. Diffusive grains

In the case where the centres of the grains diffuse randomly, the jump distribution is

$$f_\tau(\mathbf{j}) = \frac{1}{(4\pi D\tau)^{3/2}} \exp\left[-\frac{|\mathbf{j}|^2}{4D\tau}\right] \tag{S-4}$$

With this specific form, the μ-dependence in the integrand of Equation (S-3) is simply an exponential. Integrating analytically over μ leads to

$$K(r,\tau) = \frac{1}{r\sqrt{4\pi D\tau}} \int_0^\infty \rho \mathrm{d}\rho \, K(\rho) \left(\exp\left[-\frac{(\rho-r)^2}{4D\tau} \right] - \exp\left[-\frac{(\rho+r)^2}{4D\tau} \right] \right) \tag{S-5}$$

This can be conveniently written as

$$K(r,\tau) = K_a(r,\tau) + K_a(-r,\tau) \tag{S-6}$$

with

$$K_a(r,\tau) = \frac{1}{r\sqrt{4\pi D\tau}} \int_0^{2R} \rho \mathrm{d}\rho \left(1+\frac{\rho}{4R}\right)\left(1-\frac{\rho}{2R}\right)^2 \exp\left[-\frac{(\rho-r)^2}{4D\tau} \right] \tag{S-7}$$

where we have replaced $K(\rho)$ by its expression for spherical particles with radius R (Equation 9 of the main text) and replaced the upper integration bound accordingly.

In terms of dimensionless variables

$$\bar{r} = \frac{r}{R} \quad \bar{\tau} = \frac{2D\tau}{R^2} \tag{S-8}$$

the function $K_a(r,\tau)$ can be calculated as

$$\begin{aligned} K_a(\bar{r},\bar{\tau}) &= \left\{ I_0\left[\frac{2-\bar{r}}{\sqrt{\bar{\tau}}}\right] - I_0\left[\frac{-\bar{r}}{\sqrt{\bar{\tau}}}\right] \right\} \left(1 - \frac{3}{4}\bar{r} + \frac{1}{16}\bar{r}^3\right) \\ &+ \left\{ I_1\left[\frac{2-\bar{r}}{\sqrt{\bar{\tau}}}\right] - I_1\left[\frac{-\bar{r}}{\sqrt{\bar{\tau}}}\right] \right\} \left(1 - \frac{3}{2}\bar{r} + \frac{1}{4}\bar{r}^3\right) \frac{\sqrt{\bar{\tau}}}{\bar{r}} \\ &+ \left\{ I_2\left[\frac{2-\bar{r}}{\sqrt{\bar{\tau}}}\right] - I_2\left[\frac{-\bar{r}}{\sqrt{\bar{\tau}}}\right] \right\} \left(-\frac{3}{4}\bar{r} + \frac{3}{8}\bar{r}^3\right) \left(\frac{\sqrt{\bar{\tau}}}{\bar{r}}\right)^2 \\ &+ \left\{ I_3\left[\frac{2-\bar{r}}{\sqrt{\bar{\tau}}}\right] - I_3\left[\frac{-\bar{r}}{\sqrt{\bar{\tau}}}\right] \right\} \frac{1}{4}\bar{r}^3 \left(\frac{\sqrt{\bar{\tau}}}{\bar{r}}\right)^3 \\ &+ \left\{ I_4\left[\frac{2-\bar{r}}{\sqrt{\bar{\tau}}}\right] - I_4\left[\frac{-\bar{r}}{\sqrt{\bar{\tau}}}\right] \right\} \frac{1}{16}\bar{r}^3 \left(\frac{\sqrt{\bar{\tau}}}{\bar{r}}\right)^4 \end{aligned} \tag{S-9}$$

where we have introduced the notation

$$I_n(u) = \int_0^u x^n G[x] \mathrm{d}x \tag{S-10}$$

where

$$G(u) = \frac{1}{\sqrt{2\pi}} \exp\left[-\frac{u^2}{2} \right] \tag{S-11}$$

is the normal Gaussian distribution.

For $n = 0$ the value is

$$I_0(u) = \frac{1}{2}\mathrm{erf}\left[\frac{u}{\sqrt{2}}\right] \tag{S-12}$$

where erf is the error function, and the following orders are

$$I_1(u) = G[0] - G[u] \tag{S-13}$$

$$I_2(u) = I_0(u) - uG[u] \tag{S-14}$$

$$I_3(u) = 2G[0] - (2+u^2)G[u] \tag{S-15}$$

$$I_4(u) = 3I_0(u) - (3u+u^3)G[u] \tag{S-16}$$

$$I_5(u) = 8G[0] - (8+4u^2+u^4)G[u] \tag{S-17}$$

which are obtained by successive integrations by parts of Equation (S-10).

In the particular case where $r = 0$, Equation (S-9) is difficult to evaluate. In that case, it is easier to consider directly Equation (S-3) of the covariogram, which reduces to

$$K(0,\tau) = \frac{1}{(4\pi D\tau)^{3/2}} \int_0^\infty 4\pi\rho^2 \mathrm{d}\rho\, K(\rho) \exp\left[-\frac{\rho^2}{4D\tau}\right] \tag{S-18}$$

In dimensionless form, this is calculated as

$$K(0,\bar{\tau}) = 2I_2\left[\frac{2}{\sqrt{\bar{\tau}}}\right] - \frac{3}{2}I_3\left[\frac{2}{\sqrt{\bar{\tau}}}\right]\sqrt{\bar{\tau}} + \frac{1}{8}I_5\left[\frac{2}{\sqrt{\bar{\tau}}}\right]\sqrt{\bar{\tau}}^3 \tag{S-19}$$

with the same notations as in Equation (S-9).

1.2. Ballistic grains

In the case where the grains move ballistically, *i.e.* with constant velocity c along straight lines with isotropic directions, the distribution is

$$f_\tau(\mathbf{j}) = \frac{1}{4\pi|\mathbf{j}|^2}\delta(|\mathbf{j}| - c\tau) \tag{S-20}$$

In that case, the time-dependent covariogram is conveniently evaluated through Equation (S-2), which yields

$$K(r,\tau) = \frac{1}{2}\int_{-1}^{+1} \mathrm{d}\mu\, K(\sqrt{r^2 + (c\tau)^2 - 2rc\tau\mu}) \tag{S-21}$$

In terms of dimensionless variables

$$\bar{r} = \frac{r}{R} \quad \bar{\tau} = \frac{c\tau}{R} \tag{S-22}$$

the expression in Equation (S-21) is fully symmetric with respect to the interchanging of $\bar{r}$ and $\bar{t}$.

For the particular case of $\bar{r} = 0$ the result is

$$K(0,\bar{\tau}) = \left(1 + \frac{\bar{\tau}}{4}\right)\left(1 - \frac{\bar{\tau}}{2}\right)^2 \tag{S-23}$$

and for $\bar{\tau} = 0$

$$K(\bar{r},0) = \left(1 + \frac{\bar{r}}{4}\right)\left(1 - \frac{\bar{r}}{2}\right)^2 \tag{S-24}$$

For other values of $\bar{r}$ and $\bar{\tau}$, one has to notice that the argument of $K()$ in Equation (S-21) takes values from $|\bar{r} - \bar{\tau}|$ to $|\bar{r} + \bar{\tau}|$ for μ in the interval $(-1,1)$. Therefore, one also has

$$K(\bar{r},\bar{\tau}) = 0 \text{ if } |\bar{r} - \bar{\tau}| > 2 \tag{S-25}$$

For all other values the result is

$$K(\bar{r},\bar{\tau}) = \frac{1}{2\bar{r}\bar{\tau}}\{F(\min[2,\bar{r} + \bar{\tau}]) - F(|\bar{r} - \bar{\tau}|)\} \tag{S-26}$$

with

$$F(x) = \frac{x^2}{2}\left(1 - \frac{x}{2} + \frac{x^3}{40}\right) \quad . \tag{S-27}$$

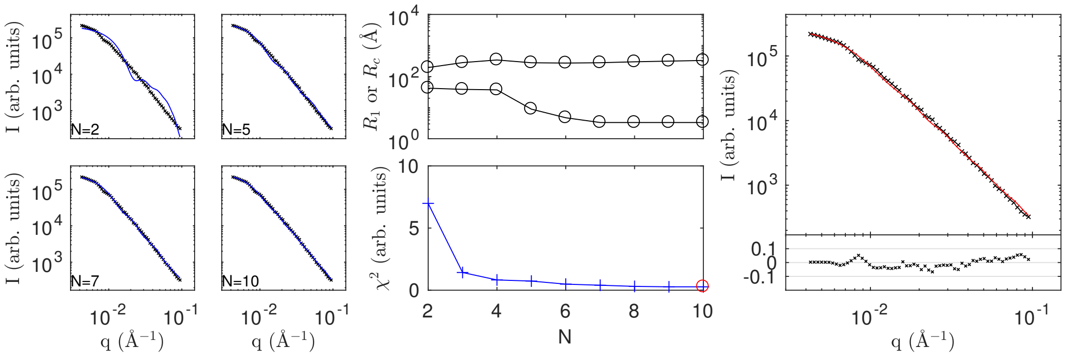

Figure S1. Fitting of the SANS of the acid-catalyzed aerogels (sample A in Figure 8). Left: least-square fits with imposed values of N and adjustable R_1 and R_c. Middle: fitted values of R_1 and R_c (top) and the corresponding χ^2 (bottom) as a function of the imposed value of N. The lowest χ^2 is circled in red. Right: least-square fit with the value of N that minimizes χ^2, with the corresponding residual (bottom). In the left and right panels, the crosses are the data and the lines are the model.

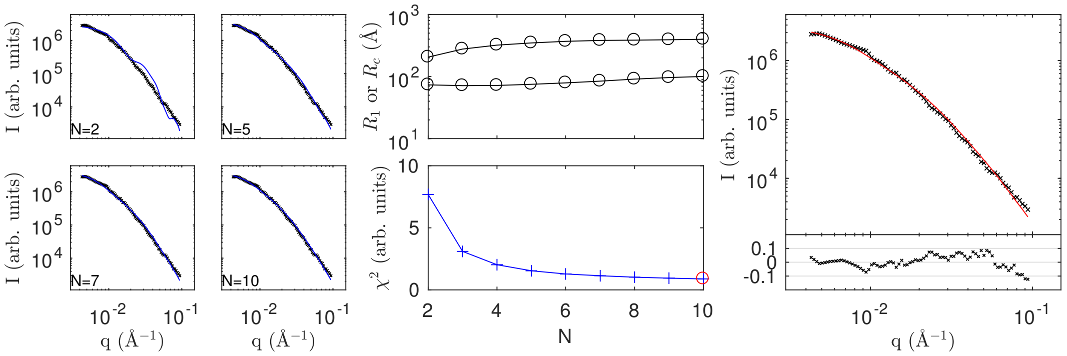

Figure S2. Fitting of the SANS of the acid-catalyzed aerogels (sample B in Figure 8). Left: least-square fits with imposed values of N and adjustable R_1 and R_c. Middle: fitted values of R_1 and R_c (top) and the corresponding χ^2 (bottom) as a function of the imposed value of N. The lowest χ^2 is circled in red. Right: least-square fit with the value of N that minimizes χ^2, with the corresponding residual (bottom). In the left and right panels, the crosses are the data and the lines are the model.

2. Fitting of SANS data with the self-similar model

We here explain in detail the fitting procedure of the SANS data with the self-similar (fractal) model, underlying Figure 8 of the main text.

The porosity of the aerogels is known from independent measurements (nitrogen adsorption), which is equivalent to knowing the overall solid fraction of the model ϕ_1. The parameters of the model remaining to be determined from the fitting of the SANS data are the upper and lower cut-offs R_1 and R_c, as well as the number of components to the hierarchical structure N.

As parameter N can only take integer values, it would not be convenient to treat it on the same footing as the other variables. A better approach consists in considering successively all values of N is a given interval, say from $N = 2$ to $N = 10$. For each value of N, the volume fraction of each hierarchical component is determined as $\varphi_1 = \phi_1^{1/N}$, and the fitting of the entire model is then done with R_1 and R_c as only adjustable parameters. The procedure is illustrated with Figures S-1 and S-2 for the acid-catalyzed and two-step acid-base aerogels of the main text, respectively.
